# Dr. Charles S. Albertson

**Published:** 1915-02

**Authors:** 


**Dr. Charles S. Albertson,** Homoeopathic Medical College of Cleveland, 1878; died at his home in Oswego, December 7, aged 62. He was born in Rush, N. Y., and practiced in Buffalo for several years. His health had been bad for several years. Appropriate resolutions were adopted by the Oswego Academy of Medicine.

